# Characterization of Photosynthetic Performance during Senescence in Stay-Green and Quick-Leaf-Senescence *Zea mays* L. Inbred Lines

**DOI:** 10.1371/journal.pone.0042936

**Published:** 2012-08-10

**Authors:** Zishan Zhang, Geng Li, Huiyuan Gao, Litao Zhang, Cheng Yang, Peng Liu, Qingwei Meng

**Affiliations:** 1 State Key Lab of Crop Biology, Tai’an, Shandong Province, China; 2 College of Life Sciences, Shandong Agricultural University, Tai’an, Shandong Province, China; 3 College of Agriculture, Shandong Agricultural University, Tai’an, Shandong Province, China; Centro de Investigación y de Estudios Avanzados del IPN, Mexico

## Abstract

The net photosynthetic rate, chlorophyll content, chlorophyll fluorescence and 820 nm transmission were investigated to explore the behavior of the photosynthetic apparatus, including light absorption, energy transformation and the photoactivities of photosystem II (PSII) and photosystem I (PSI) during senescence in the stay-green inbred line of maize (*Zea mays*) Q319 and the quick-leaf-senescence inbred line of maize HZ4. The relationship between the photosynthetic performance and the decrease in chlorophyll content in the two inbred lines was also studied. Both the field and laboratory data indicated that the chlorophyll content, net photosynthetic rate, and the photoactivities of PSII and PSI decreased later and slower in Q319 than in HZ4, indicating that Q319 is a functional stay-green inbred line. In order to avoid the influence of different development stages and environmental factors on senescence, age-matched detached leaf segments from the two inbred lines were treated with ethephon under controlled conditions to induce senescence. The net photosynthetic rate, light absorption, energy transformation, the activities of PSII acceptor side and donor side and the PSI activities decreased much slower in Q319 than in HZ4 during the ethephon-induced senescence. These results suggest that the retention of light absorption, energy transformation and activity of electron transfer contribute to the extended duration of active photosynthesis in Q319. Although the chlorophyll content decreased faster in HZ4, with decrease of chlorophyll content induced by ethephon, photosynthetic performance of Q319 deteriorated much more severely than that of HZ4, indicating that, compared with Q319, HZ4 has an advantage at maintaining higher photosynthetic activity with decrease of chlorophyll although HZ4 is a quick-leaf-senescence inbred line. We conclude that attention should be paid to two favorable characteristics in breeding long duration of active photosynthesis hybrids: 1) maintaining more chlorophyll content during senescence and 2) maintaining higher photosynthetic activity during the loss of chlorophyll.

## Introduction

To meet the demand for food for the growing world population, a significant increase in the world grain production is required, particularly in crops grown in developing countries. Historically, the increase of grain production has resulted from increase of the ratio of the grain to the total above-ground biomass (i.e., the harvest index) [1], despite little increase in the total biomass [2]. However, the harvest index of many crops is considered to be approaching a maximum [1], and further increases in yield potential may therefore require an increase in crop biomass [3]. In other words, an increase in the total net photosynthesis across the whole developmental stage is required.

Later developmental stages are the key period for the grain yield of many crops. However, the photosynthesis in leaves, especially in leaves of quick-leaf-senescence genotypes, begins to decrease during the later developmental stages, which severely limits grain yield [4], [5]. Spano et al. [3] reported that extending the duration of active photosynthesis will elevate the yield of crops and that delaying leaf senescence is one of the ways to accomplish this. A study showed that a maize hybrid with a long duration of active photosynthesis produced 24% more dry matter and assimilated 20% more nitrogen than a quick-leaf-senescence hybrid during grain filling stage [6]. In *Lolium temulentum*, it was calculated that delaying the onset of senescence by only two days could result in an increase in carbon fixation of about 11% [7]. A similar phenomenon was also observed in tobacco (*Nicotiana tabacum*) and sorghum (*Sorghum bicolor* L.) [8], [9]. The elevation of grain production in hybrids with a long duration of active photosynthesis might be much more obvious under stress conditions than under normal conditions [5], [9]. Previously, researchers have tried to extend the stay-green duration to extend the duration of active photosynthesis and breed some stay-green genotypes [6]. However, not all of the stay-green genotypes have resulted in increased grain production. The photosynthetic rate in some of the stay-green genotypes decreases at the normal rate, although the chlorophyll content decreases much slower or later than traditional hybrids. This type of stay-green genotype is denominated as a “non-functional stay green genotype”, whereas, those genotypes with both a delayed loss of pigment and a decrease in the photosynthetic rate are denominated as “functional stay-green genotypes” [10]. However, the relationship between the retention of the photosynthetic rate and the retention of the chlorophyll content has not clearly been known yet.

**Figure 1:**
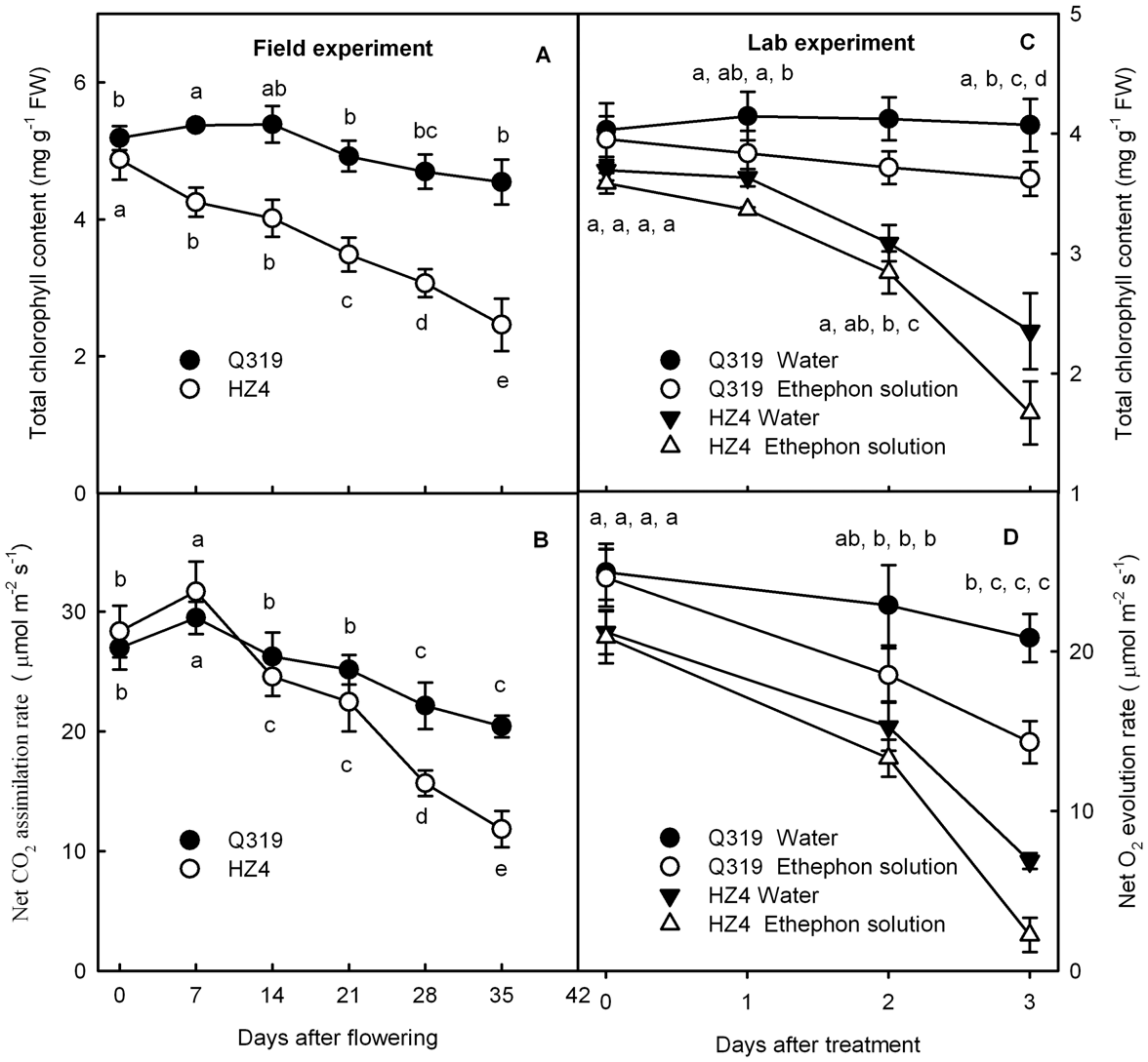
Chlorophylls content and photosynthetic rate of plants during senescence in field experiments and laboratory experiments. Total chlorophylls content (A) and net CO_2_ assimilation rate (B) in leaves of two inbred lines of maize (Q319 and HZ4) grown in the field after flowering. Total chlorophylls content (C) and net O_2_ evolution rate (D) in the detached leaf segments from the two inbred lines of maize (Q319 and HZ4) after treatment with 0.7 mmol L^−1^ ethephon or water for different days. Means±SE of six replicates are presented. Different letters indicate significant differences between the parameters in different days after flowering and ethephon treatments,, P<0.05. The differences was analyzed by LSD (least significant difference).

It has been reported that CO_2_ assimilation decreases during senescence because of non-stomatal limitation which is accompanied by the decrease in the content of soluble protein and the activities of enzymes related to the Calvin cycle [11], [12]. However, it is not enough to prove that the decrease in the activities of Calvin cycle enzymes is the cause of the decreased CO_2_ assimilation because ATP and NADPH produced via the photosynthetic electron transfer are also essential for CO_2_ assimilation. It is known that under some stress conditions, decay in light absorption, energy transformation and electron transfer will result in a decrease in ATP and NADPH production, which limits photosynthesis [13]–[18]. This was confirmed by studies of photosynthetic electron transfer carrier mutants, which have lower photosynthetic rate, lower electron transport rate (ETR) and lower growth rate [19], [20]. Senescence can be regarded as a kind of stress for plants. Proteins, especially the proteins in thylakoid membrane, can be damaged during senescence even in the dark or under low irradiation [11], [21]–[23]. The majority of previous studies have focused on the changes in the chlorophyll content, RNA and protein expression in leaves during senescence, especially the content of the protein complex in thylakoid membranes and enzymes related to the Calvin cycle [11], [21]–[25]. However, the changes in the activities of light absorption, energy transformation and electron transfer during senescence remain unclear, partially due to a lack of instruments that can perform in vivo measurements. PEA-senior (now named as M-PEA), a new product of Hansatech (UK) can simultaneously measure chlorophyll a fluorescence transient and 820 nm transmission to examine the activities of PSII and PSI. The high time resolution detection for the discrimination of fast fluorescence induction kinetics makes it possible to investigate the activity of the light reaction, especially the activity of primary reaction in vivo. The PEA-senior has been widely used to study the activities of PSII and PSI, especially the interaction between the 2 systems under stress conditions [26]–[31].

We sought to address the following questions: In addition to the difference in degradation of the chlorophyll content, are there any differences in the light absorption, energy transformation and electron transfer activity during senescence of different stay-green genotypes of maize? What is the relationship between the decrease in the photosynthetic performance and the decrease in the chlorophyll content? Are the relationships different between different stay-green genotypes of maize? Addressing these questions will provide agricultural scientists with useful information for the breeding of hybrids with a long duration of active photosynthesis.

## Materials and Methods

### Plant Materials

Two inbred lines of maize were used in this experiment: stay-green inbred line Qi-319 (Q319) and quick-leaf-senescence inbred line Huangzao-4(HZ4).

In the field experiment, plants were grown on a farm of Shandong Agriculture University at June of 2009. Nutrients and water were supplied sufficiently throughout to avoid any potential nutrient and drought stresses. The net photosynthetic rates and chlorophyll contents of the ear leaves were measured every 7 days after flowering that is defined as half of the pollen being shed.

In the laboratory experiment, plants were grown in 37 cm diameter pots. Nutrients and water were supplied sufficiently throughout to avoid any potential nutrient and drought stresses. The pots were placed in a growth cabinet maintained at 25°C during the light period (600 µmol m^−2^ s^−1^, 16 h photoperiod) and 22°C during the 8 h dark period. Leaf segments with 15 cm length from the middle of the third leaves from the top of plants that were approximately 8 week old were used for experiments. The basal part of the leaf segments were dipped into 0.7 mmol L^−1^ ethephon (Sigma, U.S.A) solution or water under 15 µmol m^−2^ s^−1^ light at 25°C. The ethephon solution and water were exchanged every day.

### Measurement of the Chlorophyll Content

Leaf chlorophyll was extracted with 80% acetone in the dark for 72 h at 4°C. The extracts were analyzed using an UV-visible spectrophotometer UV-1601 (Shimadzu, Japan) according to the method of Porra [32].

### Measurement of the Net CO_2_ Assimilation Rate

The net CO_2_ assimilation rate was measured with a CIRAS-2 portable photosynthesis system (PP Systems, U.S.A). The CO_2_ concentration, relative humidity, photon flux density (PFD) and leaf temperature for all measurements were maintained at 360 µmol mol^−1^, 80%, 1600 µmol m^−2^ s^−1^ and 25°C via an automatic control device of the CIRAS-2 photosynthesis system.

### Measurement of the Net O_2_ Evolution Rate

A Chlorolab-2 liquid-phase oxygen electrode system (Hansatech, UK) was used to measure the net O_2_ evolution rate of leaf segments in 50 mM NaHCO_3_ solution (dissolved in 50 mM Tris–HCl buffer, pH 7.5) at 25°C. A photosynthetic saturation light (1600 µmol m^−2^ s^−1^) was used in the measurements.

### Measurements of Chlorophyll a Fluorescence Transient and 820 nm Transmission

The chlorophyll a fluorescence transient and the 820 nm transmission changes were simultaneously measured using an integral PEA Senior (Hansatech, UK). The saturating red light, at 3000 µmol m^−2^ s^−1^ was produced by an array of four 650 nm light-emitting diodes (LED) (peak 650 nm), and the far-red light source was a QDDH735020 LED (Quantum Devices Inc., USA). The modulated (33.3 kHz) far-red measuring light (820 nm) was provided by an OD820 LED (Opto Diode Crop, USA). Irradiated with a far-red pulse (250 µmol m^−2^ s^−1^ PFD), the transmission at 820 nm in leaves decreases gradually, which is mainly caused by the oxidation of P700 (the primary electron donor in PSI) and plastocyanin (PC) [33]. The changes in the amplitude of the 820 nm transmission (ΔI/Io) have been widely used to estimate the activity of PSI complex in vivo [33]–[38].

Chlorophyll a fluorescence transient obtained from the dark-adapted sample was analyzed with the JIP-test [39]). The description and calculation formula of parameters were listed below.

1. Absorption flux per cross section of leaf, ABS/CSo≈Fo
2. The normalized relative variable fluorescence at the K step (WK), WK = (FK−Fo)/(FJ−Fo)
3. The relative variable fluorescence at the J step (VJ), VJ = (FJ−Fo)/(Fm−Fo)
4. The maximum quantum yield of PSII, Fv/Fm = 1−(Fo/Fm)
5. The efficiency of electron move beyond QA−, ETo/TRo = 1−VJ
6. Quantum yield for electron transport, ETo/ABS = {1−(Fo/Fm)}·ETo/TRo
7. Density of active reaction centers (QA-reducing PSII reaction centers), RC/CSo = (Fv/Fm)·(VJ/Mo)·(ABS/CSo)

### Statistical Analysis

LSD (least significant difference) was used to analyze differences between the different treatments by using SPSS 16.

**Figure 2:**
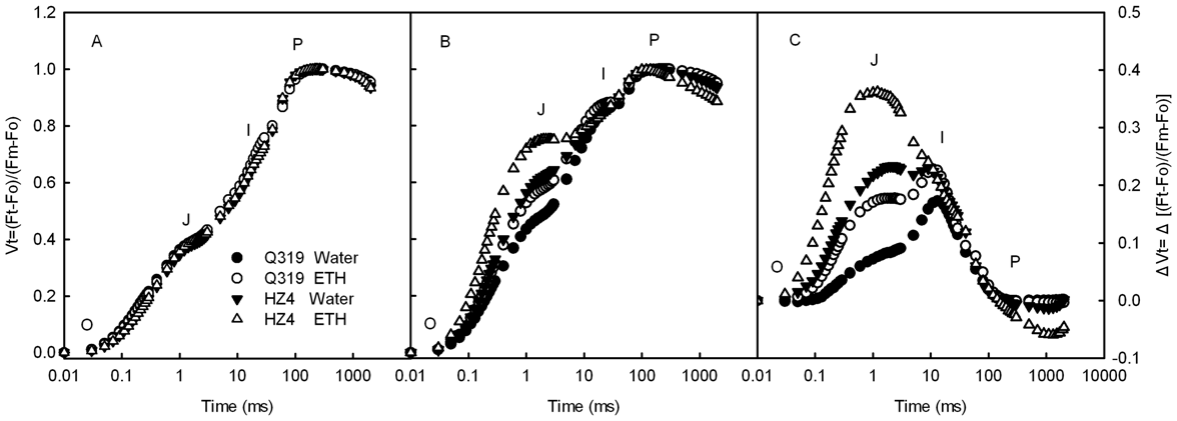
Chl a fluorescence transients normalized between Fo to F_P_ in detached leaf segments during senescence. Chl a fluorescence transients in detached leaf segments from the two inbred lines of maize (Q319 and HZ4) before the treatment (A), and after 3 days of treatment with 0.7 mmol L^−1^ ethephon or water (B). Chl a fluorescence transients were normalized between Fo to F_P_ (Vt = (Ft-Fo)/(Fm–Fo)) (A, B). ΔVt (C) was obtained by subtracting the kinetics of leaf segments before treatment from the kinetics of leaf segments 3 days after treatment. O indicates the O step at about 20 µs; J indicates the J step at about 2 ms; I indicates the I step at about 30 ms; P indicates the P step, the maximum fluorescence. (Each datum is the average of 6 independent measurements.).

## Results

### Changes in the Chlorophyll Content and the Net CO_2_ Assimilation Rate during Senescence

The chlorophyll content and the net CO_2_ assimilation rate in leaves of the two inbred lines of maize grown in the field decreased continuously 7 days after flowering (Fig. 1). However, extents of the decreases of both chlorophyll content and net CO_2_ assimilation rate in Q319 were significantly less than those in HZ4 (P<0.05), which indicates that Q319 is a functional stay-green genotype.

**Figure 3:**
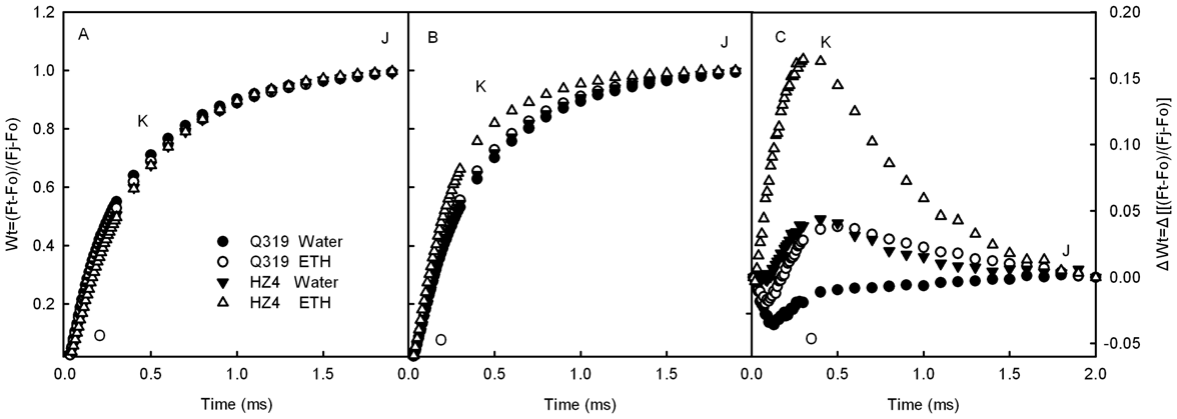
Chl a fluorescence transients normalized between Fo to F_J_ in detached leaf segments during senescence. Chl a fluorescence transients in leaf segments from the two inbred lines of maize (Q319 and HZ4) before treatment (A), and after 3 days of treatment with 0.7 mmol L^−1^ ethephon or water (B). Chl a fluorescence transients were normalized between Fo to F_J_ (Wt = (Ft–Fo)/(F_J_–Fo)) (A, B). ΔWt (C) was obtained by subtracting the kinetics of the leaf segments before treatment from the kinetics of leaf segments 3 days after treatment. O indicates the O step at about 20 µs; K indicates the K step at about 300 µs; J indicates the J step at about 2 ms. (Each datum is the average of 6 independent measurements.).

### Changes in the Chlorophyll Content during Ethephon-induced Senescence

To avoid the influence of difference in development stages of the two inbred lines and fluctuation of environment factors on the changes of the photosynthetic activity and the chlorophyll content, ethephon was used to induce senescence of age-matched detached leaf segments under controlled conditions. Ethephon releases ethylene and enhances ethylene concentration in plants. Ethylene is known to accelerate senescence in mature leaves [40]. The chlorophyll content in the detached leaf segments of the Q319 controls did not change detectably after having been detached from plants for 3 days. The chlorophyll content in the ethephon-treated leaf segments decreased slightly (<10%) compared with the control. However, the chlorophyll contents in detached leaf segments of HZ4 in both the ethephon treatment and the control decreased markedly (P<0.05) after detachment from plants for only two days (Fig. 1). The chlorophyll content in the leaf segments treated with ethephon decreased more severely than that of the control: three days after treatment, the chlorophyll content decreased by 36.3% and 53.5% in the control and in the ethephon-treated leaf segments, respectively.

**Figure 4:**
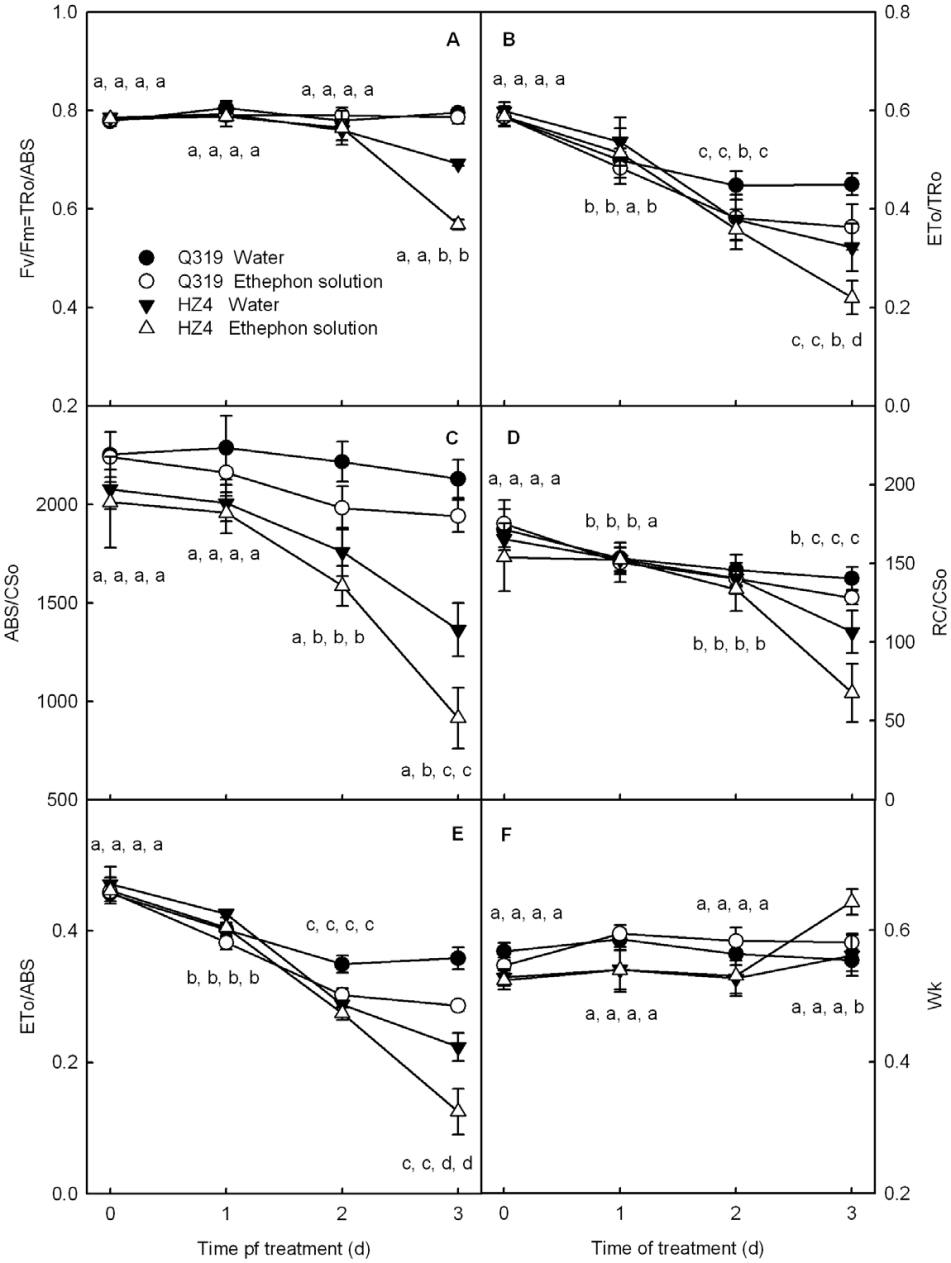
PSII performance obtained by JIP-text in detached leaf segments during senescence. The maximum quantum yield of PSII (Fv/Fm) (A), the efficiency of electron move beyond Q_A_^−^ (ETo/TRo, B), the absorption flux per CS (ABS/CSo, C), the density of Q_A_^−^ reducing PSII reaction centers (RC/CSo, D), quantum yield for electron transport further than Q_A_^−^ (ETo/ABS, E) and normalized relative variable fluorescence at the K step (W_K_, F) in leaf segments from the two inbred lines of maize (Q319 and HZ4) treated with 0.7 mmol L^−1^ ethephon or water for different days. The means±SE of six replicates are presented. Different letters indicate significant differences between the parameters in different days after ethephon treatments, P<0.05. The differences was analyzed by LSD (least significant difference).

### Changes in the Photosynthetic O_2_ Evolution during Ethephon-induced Senescence

The net O_2_ evolution rate in all leaf segments decreased significantly (P<0.05) two days after detachment from plants (Fig. 1). The extent of the decreases of net O_2_ evolution rate in both the control and ethephon-treated HZ4 leaf segments was much greater than that in Q319. However, ethephon treatment enhanced the extent of the decreases in both Q319 and HZ4.

### Changes in the Chl a Fluorescence Transients during Ethephon-induced Senescence

All chlorophyll a fluorescence transients showed a typical polyphasic rise with the basic steps of O-J-I-P. The chlorophyll a fluorescence transients (Vt curve) of all the detached leaf segments were similar before ethephon treatment (Fig. 2A). The J (2 ms) and I (30 ms) steps in the chlorophyll a fluorescence transients of both that Q319 and HZ4 leaf segments increased markedly after 3 days of treatment (Fig. 2B). The amplitudes of the J and I steps were greatly increased by ethephon treatment. It was noticed that, after 3 days of treatment, the most distinct peaks in the ΔVt curves of HZ4 appeared at the J step (Fig. 2C), and the relative variable fluorescence at the J step in HZ4 leaf segments increased much more markedly than in Q319 leaf segments (Fig. 2 B, C). In contrast, the most distinct peaks in the ΔVt curves of Q319 appeared around the I step (Fig. 2C). The appearance of the peak at J step indicates that electron transport beyond Q_A_^−^ was limited [41]–[43], and the appearance of the peak at I step indicates that electron transport from plastoquinone (PQ) to PSI acceptor side was limited [43]–[46].

**Figure 5:**
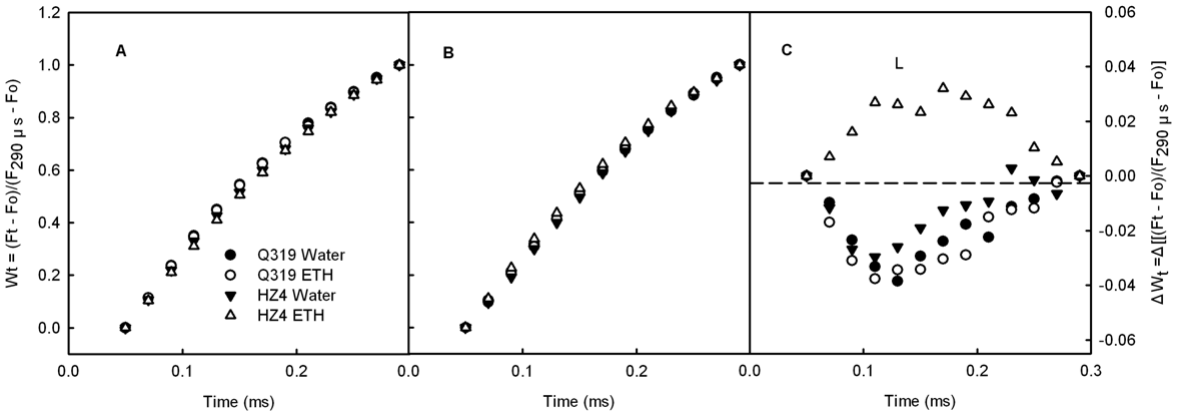
Chl a fluorescence transients normalized between Fo to F_290µs_ in detached leaf segments during senescence. Chl a fluorescence transients in leaf segments from the two inbred lines of maize (Q319 and HZ4) before treatment (A), and after 3 days of treatment with 0.7 mmol L^−1^ ethephon or water (B). Chl a fluorescence transients were normalized between Fo to F_290µs_ (Wt = (Ft–Fo)/(F_290µs_–Fo)) (A, B). ΔWt (C) was obtained by subtracting the kinetics of the leaf segments before treatment from the kinetics of leaf segments 3 days after treatment. L indicates the L-band at about 130 µs. (Each datum is the average of 6 independent measurements.).

The K step of the Chl a fluorescence transients (at 300 µs) of both Q319 and HZ4 increased markedly after 3 days of treatment. The amplitude of K step in the HZ4 leaf segments treated with ethephon was the greatest (Fig. 3). The W_K_ also exhibited a similar change (Fig. 4F). The increases in the K step fluorescence and W_K_ have been widely used as specific indicators of injury to the oxygen evolving complex (OEC), in other words, as specific indicators of PSII donor photohibition [41], [47], [48]. Our observations indicate that the harm to the PSII donor side was more severe in HZ4 than in Q319 during ethephon-induced senescence.

As shown in Fig. 5, the L-band in the HZ4 detached leaf segments treated with ethephon was positive, but the L-bands in other leaf segments were negative (Fig. 5C). According to the Grouping Concept [49] and JIP-test [17], [50], the positive L-band indicates that the PSII units were less grouped or that less energy was exchanged between independent PSII units. Because the grouped conformation is more stable than the ungrouped one [17], [50], the decreased grouping indicates that the PSII units had lost their stability and become more fragile. This observation implies that the harm to the PSII units in HZ4 was more severe than that in Q319 during the ethephon-induced senescence.

**Figure 6:**
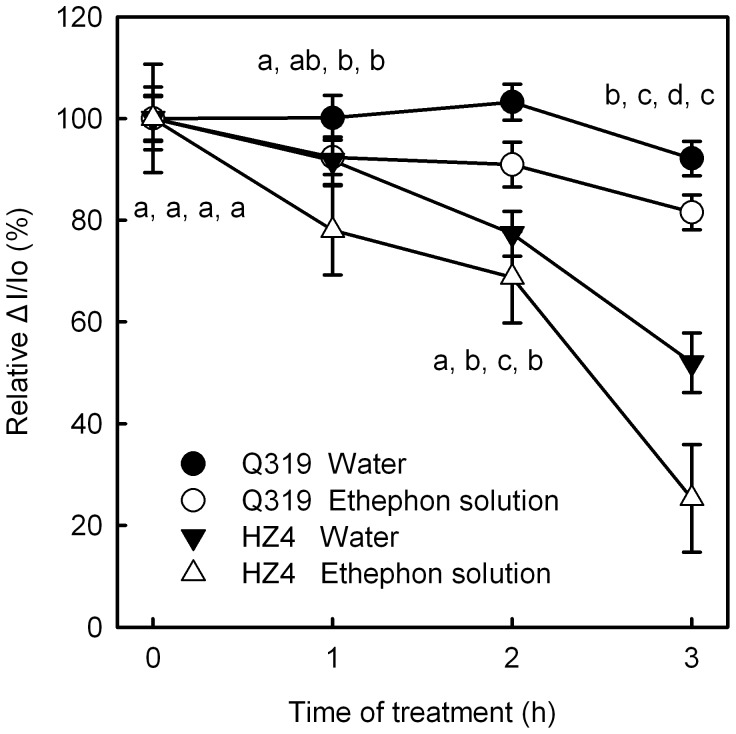
ΔI/Io in detached leaf segments during senescence. The change in the amplitude of 820 nm transmission (ΔI/Io) in leaf segments from the two inbred lines of maize (Q319 and HZ4) treated with 0.7 mmol L^−1^ ethephon or water for different days. The initial values of ΔI/Io before treatment were taken as 100%, whereas those after treatment were taken as the percentage of the initial values. The means±SE of six replicates are presented. Different letters indicate significant differences between the parameters in different days after flowering and ethephon treatments, P<0.05. The differences was analyzed by LSD (least significant difference).

### The Activity of PSII during Ethephon-induced Senescence

To further investigate the changes in light absorption, energy transformation and PSII photoactivities in the two inbred lines of maizes during senescence, the JIP-test was used to analyze the chlorophyll a fluorescence transients. The efficiency of electron moves beyond QA^−^ (ETo/TRo), which reflects the probability for electron transport further than Q_A_^−^ (the photoactivity of PSII acceptor side) decreased significantly (P<0.05) after treatment in all detached leaf segments (Fig. 4B). The ETo/TRo in HZ4 leaf segments decreased more severely than that in Q319 leaf segments, and the ETo/TRo in leaf segments treated with ethephon decreased more severely than that in controls. Absorption flux per cross section of leaf (ABS/CSo) and the density of Q_A_-reducing PSII reaction centers (RC/CSo) changed similarly to ETo/TRo (Fig. 4C, D).

**Figure 7:**
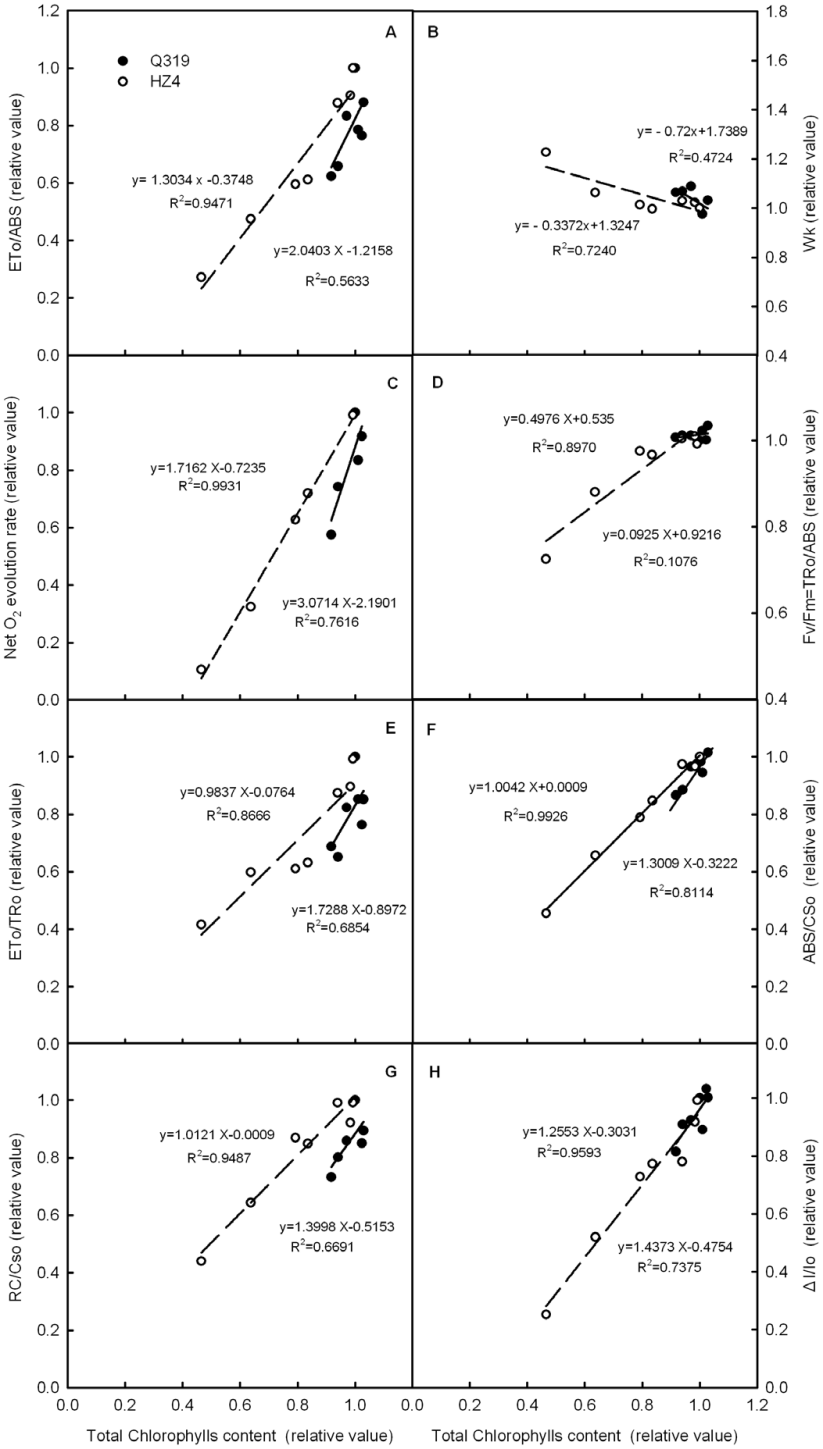
The relationship between chlorophyll content and photosynthetic performance in leaf segments during senescence. Relationships between the quantum yield for electron transport further than Q_A_^−^ (ETo/ABS) (A), W_K_ (B), net O_2_ evolution rate (C), maximum quantum yield of PSII (Fv/Fm = TRo/ABS) (D), efficiency of electron move beyond Q_A_^−^ (ETo/TRo) (E),absorption flux per cross section of leaf (ABS/CSo) (F), density of Q_A_^−^reducing PSII reaction centers(RC/CSo) (G), maximum PSI redox activity (ΔI/Io) (H) and leaf chlorophyll content in leaf segments treated with 0.7 mmol L^−1^ ethephon or water. “•” and “○” represent the stay-green inbred line of maize Q319 and the quick-leaf-senescence inbred lines of maize HZ4, respectively. The initial values of all the parameters in leaf segments before treatment were taken as 1, whereas those after treatment were taken as the proportion of the initial values. (Each datum is the average of 6 independent measurements.).

The maximum quantum yield of PSII (Fv/Fm) in detached leaf segments of HZ4 treated with ethephon or water decreased by 27.5% and 11.9%, respectively, after 3 days of treatment (Fig. 4A). However, Fv/Fm in detached leaf segments of Q319 was maintained unchanged throughout the period of treatment with ethephon or water (Fig. 4A).

### The Activity of PSI during Ethephon-induced Senescence

The change in the amplitude of 820 nm transmission (ΔI/Io) has been widely used to estimate the activity of PSI complex in vivo [33]–[38]. The ΔI/Io in detached leaf segments of HZ4 treated with ethephon or water decreased along with the time, the ΔI/Io in leaves treated with ethephon or water decreased by 74.7% and 48.0% after 3 days of treatment, respectively (Fig. 6). However, the ΔI/Io in detached leaf segments of Q319 treated with ethephon or water decreased by only 18.5% and 7.9% after 3 days of treatment, respectively.

### The Relationship between the Chlorophyll Content and the Photosynthetic Performance in Leaf Segments

To further investigate the relationship between the chlorophyll content and the photosynthetic performance in leaves of the two inbred lines of maize, we analyzed the changes of net O_2_ evolution rate, photoactivities of PSII and PSI on the basis of chlorophyll content in the leaves (Fig. 7). We observed that the W_k_ in leaves of the both inbred lines increased with decreasing of chlorophyll content, whereas the ETo/ABS, net O_2_ evolution rate, Fv/Fm, ETo/TRo, ABS/CSo, RC/CSo and ΔI/Io in the leaves of both inbred lines decreased with decreasing chlorophyll content. However, all of the above-mentioned parameters changed faster in Q319 leaf segments than in HZ4, which indicates that with a similar decrease in the chlorophyll content, the net photosynthetic rate, light absorption and photosynthetic electron transfer activity decreased more severely in Q319 than in HZ4. The Fv/Fm in leaf segments of Q319 remained unchanged with decreasing chlorophyll content.

## Discussion

Only according to the later and slower decrease of chlorophyll content at the later developmental stage in Q319, we could not confirm that Q319 has a longer duration of photosynthetic activity. However, the fact that the decrease in both net CO_2_ assimilation rate and chlorophyll content in the field-grown Q319 was much slower than that in HZ4 (Fig. 1B) demonstrates that Q319 is a functional stay-green inbred line. To avoid the difference in development stages between Q319 and HZ4 and the fluctuation in environmental factors influencing the results, senescence in leaves from Q319 and HZ4 was induced by ethephon under precisely controlled conditions. The observation that the net O_2_ evolution rate, light absorption, energy transformation and photoactivities of PSII and PSI were higher in Q319 than in HZ4 during senescence under the control conditions (Fig. 2) supports the conclusion that Q319 is a functional stay-green inbred line. Thomas and Howarth [51] defined five stay-green types according to the decrease of chlorophyll content during senescence, the type A and B stay-green differ from the normal phenotype by later onset of senescence and slower senescence rate, respectively. The decrease in the chlorophyll content was later and slower in Q319 than in HZ4 leaves, (Fig. 1), indicating that Q319 has characteristics of both A and B stay-green types.

Considerable efforts have been directed toward elucidating the mechanism of functional stay-green [12], [50]–[54]. However, few works have focused on the changes in light absorption, energy transformation and electron transfer during senescence. Our study showed that significant differences of light absorption, energy transformation and electron transfer existed between the stay-green and the quick-leaf-senescence inbred lines during senescence. First, the light absorption indicated by ABS/CSo decreased faster during senescence in the quick-leaf-senescence inbred line than in the stay-green line (Fig. 4). Second, the energy transformation indicated by Fv/Fm decreased obviously in leaves of the quick-leaf-senescence inbred line but remained stable in leaves of the stay-green line during senescence (Fig. 4). Third, the photosynthetic electron transfer capacity declined faster in the quick-leaf-senescence inbred line than in the stay-green line during senescence , which was indicated by the faster decreases of the density of active PSII reaction centers( RC/CS), the O_2_ evolution rate and the PSI photoactivity (ΔI/Io) in HZ4 than in Q319 (Fig. 4, 6).

It is well known that inactivation of light absorption, energy transformation and photosynthetic electron transfer will reduce the production of ATP and NADPH, hence limiting CO_2_ assimilation [18], [55]. Although it has been reported that the total soluble protein content, especially enzymes in Calvin cycle such as Rubisco decreased markedly during senescence [11], [56], it is hard to determine whether the decrease in the activities of the enzymes is the key factor that results in the decrease in photosynthesis under different conditions, because the decreases in production of ATP and NADPH and RuBP regeneration due to the decrease in light absorption, energy transformation and electron transfer activity during senescence might also affect CO_2_ assimilation. In addition, Wingler et al [56] reported that, during senescence, the soluble protein content and activities of key enzymes in the Calvin cycle were much higher in a transgenic stay-green tobacco plant than in the WT, but the photosynthetic rate in the transgenic tobacco plant was only slightly higher than that in the WT. From this, the authors inferred that the activity of the enzymes in Calvin cycle is not the only factor that dominates photosynthesis during senescence [56]. Together with our results, we suggest that the faster decay of light absorption, energy transformation and electron transfer activities might be one of the important reasons for the faster decrease of photosynthesis in the quick-leaf-senescence inbred line HZ4.

Though in both the field and controlled conditions, the chlorophyll content, photosynthesis and photosynthetic performance decreased much slower and later in the stay-green inbred line Q319 than in the quick-leaf-senescence inbred line HZ4 (Fig. 1, 4, 6), when an identical decrease in chlorophyll content was induced by ethephon, the photosynthetic performance was deteriorated much more severely in the stay-green inbred line Q319 than in the quick-leaf-senescence HZ4 (Fig. 7). These data demonstrated that although HZ4 is a quick-leaf-senescence inbred line, it has an advantage at maintaining photosynthetic activity with a decrease in chlorophyll. This characteristic in HZ4 might be an acclimation strategy to the severe loss of chlorophyll during senescence. Although it is known that maintaining chlorophyll is not significant for maintaining photosynthesis when the activities of enzymes related to the Calvin cycle decrease severely during senescence, it is the first time that our study brought out that the maintenance of photosynthetic performance during senescence depends not only on stay green but also on the ability of maintaining high light absorption, energy transformation and electron transfer.

These results will provide useful information for agricultural scientists in breeding crops with a long duration of active photosynthesis. We suggest that the two characteristics: 1) maintaining more chlorophyll content during senescence and 2) maintaining higher light absorption, energy transformation and electron transfer with the decrease in chlorophyll, should be considered to be important factors in the breeding of new breeds. However, during senescence, what is the specific mechanism of decrease in the photosynthetic rate and the chlorophyll content in different kinds of inbred lines? How is the mechanism regulated, and what is the limiting step of the decrease in photosynthesis during senescence? Further cooperative studies from different fields are needed to address these questions.
